# Beyond clinical data: the role of sewage monitoring in tracking pandemic trends of SARS-CoV-2

**DOI:** 10.3389/fmicb.2025.1657397

**Published:** 2025-08-19

**Authors:** Zhenlu Sun, Yulou Sun, Kai Guo, Lili Zhao, Cong Li, Yi Zhang, Shi Cui Yan, Jian Yang, Guifang Zhang

**Affiliations:** Yantai Center for Disease Control and Prevention, Yantai, China

**Keywords:** SARS-CoV-2, sewage, RT-qPCR, high-throughput sequencing, genomic surveillance

## Abstract

**Introduction:**

The SARS-CoV-2 pandemic has caused a global crisis that has impacted not only health care systems, but also economies and societies. The constraints in clinical testing provide challenges in reliably assessing the prevalence of variations, particularly in regions with limited resources, testing, and sequencing capabilities. Sewage-based epidemiology uses SARS-CoV-2 in sewage as an indicator, can monitor and provide early warning of viral transmission in communities, thereby informing response strategies.

**Methods:**

In this study, sewage samples and clinical patient samples were collected in Yantai City, Shandong Province. RT-qPCR and high-throughput sequencing techniques were employed to identify and analyze SARS-CoV-2, respectively.

**Results:**

Our results showed that the dynamic trend of SARS-CoV-2 RNA concentration in sewage samples coincided with the positive rate of clinical surveillance cases (Spearman’s *ρ* = 0.97, *p* < 0.001). A significantly higher number of SARS-CoV-2 lineages were detected in sewage compared to clinical samples (paired *t*-test, *t* = 6, df = 4, *p* < 0.05), and the growth of the dominant strain can be detected in sewage samples up to a week in advance.

**Discussion:**

Our study demonstrates that effluent genomic surveillance is a rapid, sensitive, and scalable method. It enables the timely identification of SARS-CoV-2 variants and the detection of hidden transmission. It can be applied to SARS-CoV-2 early warning as well as epidemiologic surveillance. However, this study has certain limitations. First, due to financial constraints, only a limited number of clinical samples were analyzed, which may have underestimated the diversity of SARS-CoV-2 lineages in the patient population. Second, the absence of information on the physicochemical characteristics of sewage may have limited our understanding of environmental factors affecting viral stability and detection efficiency.

## 1 Introduction

SARS-CoV-2 is the pathogen that causes COVID-19 (Zhou et al., 2020; Wu et al., 2020), which has caused more than 700 million infections and over 7 million deaths worldwide since it was first discovered in late 2019.^1^ SARS-CoV-2 continues to evolve as it spreads into new regions, resulting in a variety of novel strains with enhanced transmissibility, increased disease severity, and improved immune escape capabilities (Gangavarapu et al., 2023; Harvey et al., 2021). Timely, accurate, and thorough surveillance and detection are critical to effectively controlling and preventing the spread of SARS-CoV-2. However, surveillance of population dissemination is labor-intensive, and monitoring scattered cases may not provide a comprehensive picture of the disease’s epidemiological trends in the population. Timely tracking of molecular evolutionary variations in the virus is a challenging task. Systemic healthcare disparities, particularly in underprivileged and underserved communities, can also lead to sampling bias (Reitsma et al., 2021; Lieberman-Cribbin et al., 2020).

In contrast, PCR-based SARS-CoV-2 RNA sewage monitoring serves as a novel public health intervention. The abundance of SARS-CoV-2 RNA in sewage within a community or region can reflect the infection status of that area, regardless of individual differences, symptom presentation, willingness to test, and healthcare resources. Monitoring SARS-CoV-2 in water and sewage offers several benefits, including noninvasiveness, low cost, high coverage, early warning and trend analysis (Karthikeyan et al., 2022; Kitajima et al., 2020; Mallapaty, 2020; Mohan et al., 2021; Ahmed et al., 2020; Randazzo et al., 2020; Collivignarelli et al., 2020). Meanwhile, sequencing of viral genomes from sewage, also known as “sewage genome surveillance” can clarify epidemiologic transmission links and track viral lineages in populations (Hata et al., 2021; Karthikeyan et al., 2021; Randazzo et al., 2020). This approach has the potential to cost-effectively capture community virus transmission and elucidate variants (including emerging variants) (Karthikeyan et al., 2021; Mercer and Salit, 2021).

The classification of SARS-CoV-2 lineages relies on systems such as PANGOLIN (Phylogenetic Assignment of Named Global Outbreak Lineages), which categorize the virus into distinct branches based on characteristic patterns of genomic mutations (O’Toole et al., 2021). These lineages reflect the ongoing global genetic evolution of the virus. Systematic surveillance of SARS-CoV-2 lineages improves our understanding of viral transmission dynamics and enables the early detection of variants of concern (VOCs) with enhanced transmissibility or immune evasion capabilities, thereby offering critical insights to guide public health interventions. In recent years, sewage-based genomic surveillance has emerged as a valuable approach for monitoring the spread of SARS-CoV-2. Several studies have shown that high-throughput sequencing of viral RNA from sewage can reliably identify dominant SARS-CoV-2 lineages and, in some cases, detect emerging or low-frequency variants before they are reported through clinical surveillance (Cancela D'Angelo et al., 2023; Harrington et al., 2024; Kadam et al., 2025; Yousif et al., 2023). Despite technical challenges such as low viral loads and complex sample backgrounds, advances in high-sensitivity sequencing platforms and specialized bioinformatics tools (e.g., Freyja) have greatly improved the ability to resolve SARS-CoV-2 lineage compositions from sewage samples (Karthikeyan et al., 2022). These technological developments have significantly expanded the utility of sewage surveillance in tracking SARS-CoV-2 evolution, supporting early warning systems, monitoring transmission trends, and guiding public health responses.

With the complete relaxation of China’s SARS-CoV-2 epidemic prevention and control measures in early December 2022, the SARS-CoV-2 virus has become widely prevalent in the population, leading to an increase in the number of cases. We also detected high concentrations SARS-CoV-2 RNA in the sewage of our city. In order to grasp the prevalence and abundance changes of SARS-CoV-2 subtypes in the city, we selected 7 sewage treatment plants in the Yantai area and collected sewage samples from the 3rd to the 12th week of 2023, and used RT-qPCR and amplicon-based whole genome sequencing to detect SARS-CoV-2. We conducted a comparative analysis of sewage surveillance data and clinical surveillance data to evaluate the significance of monitoring SARS-CoV-2 in sewage.

According to our research, the copies of SARS-CoV-2 RNA in sewage may be used as an indicator of epidemic since they are closely related to the infections in the population. Large-scale genomic surveillance of sewage is feasible for early detection and tracking of viral lineages. It can assess the epidemiological intensity of SARS-CoV-2 infection outbreaks, track trends, and provide data support for outbreak research and judgment. It can be used as an early warning system by public health authorities to take appropriate action.

## 2 Materials and methods

### 2.1 Sample collection

From the 1st to the 12th week of 2023, we collected a total of 614 clinical samples from 16 sentinel hospitals for SARS-CoV-2 monitoring. This study was approved by the Ethics Committee of Yantai Center for Disease Control and Prevention (Approval No.: YYLLS 2023–16) and conducted in accordance with the Declaration of Helsinki. According to the requirements of *Shandong Province SARS-CoV-2 Virus in Urban Sewage Monitoring Work Program*, a total of 7 sewage treatment plants were selected in different districts and counties in Yantai City, China, to collect sewage samples. Since it takes a lot of time to plan and arrange sewage sampling points, the collection period was from the 3rd to the 12th week of 2023, with a total of 375 sewage samples. An automatic sampler collected 2,400 mL of mixed effluent samples per hour from the water treatment plants between 10 a.m. and 10 p.m. We sterilized the bottles with 75% alcohol and transported them to the laboratory in a refrigerated box within 2 h. The laboratory will complete the quantitative testing of the SARS-CoV-2 within 24 h after receiving the samples.

### 2.2 Samples treatment

The sewage sample was first pre-centrifuged in accordance with The centrifugal ultrafiltration method in the Standard for Methods of Enrichment Concentration and Nucleic Acid Detection of SARS-CoV-2 Virus in Sewage (WS/T799-2022) (National Health Commission of the People’s Republic of China, 2022): First, the sample was pre-centrifuged: 50 mL of sewage sample was taken in a 50 mL centrifuge tube and centrifuged at 4 °C at 5,000 g for 30 min. The supernatant and the precipitate were kept separately; 0.5 mL of the supernatant was added to the centrifuge tube containing the precipitate; the precipitate was resuspended and mixed well; and it was retained for later use. The pre-centrifuged supernatant was transferred to a sample concentration cup and centrifuged at 4 °C, 2,480 g for 15 min. If the volume of residual liquid in the sample concentration cup is large, the centrifugation time can be extended as needed until the volume of the liquid in the concentration cup is about 0.3–0.6 mL. Once the entire supernatant is concentrated, pipette the concentrate and carefully pipette the membrane several times, then all the concentrate is pipetted and transferred to a 2 mL centrifuge tube. Finally, 0.5 mL of the pre-centrifuged precipitate suspension was added, which was the concentrate of the sewage sample, and the volume was about 0.8–1.1 mL.

SARS-CoV-2 viral RNA was extracted from 140 μL specimens using the QIAamp viral RNA mini kit (Qiagen, Hilden, Germany) following the manufacturer’s protocol. The purified RNA was eluted in 50 μL elution buffer. The extracted RNA was subsequently screened for SARS-CoV-2 RNA via real-time RT–qPCR (Bioperfectus Technologies, Jiangsu, China) for two gene targets: the Open Reading Frame gene region (ORF1a/b) and Nucleocapsid region (N). A sample was considered positive if both targets were detected with a Ct value below 37. All samples with Ct values below 32 were selected for the subsequent whole viral genome sequencing.

### 2.3 Quantification of SARS-CoV-2 in sewage water

Quantitative detection of SARS-CoV-2 was as follows: A certified SARS-CoV-2 genomic RNA standard material (National Institute of Metrology, China; reference number: GBW (E)091089) was used to prepare a standard curve, consisting of six serially diluted concentration points. Based on the concentration of the standard material, it was diluted in a gradient, and triplicate measurements were made for each concentration. The Ct value was obtained by fluorescence quantitative RT-PCR, and the standard curve was plotted with the Ct value as the vertical coordinate and the logarithmic value of the concentration as the horizontal coordinate. The Ct values from sewage samples were then applied to the standard curve to calculate the SARS-CoV-2 RNA concentration (copies/mL).

### 2.4 NGS sequencing strategies

In this study, we screened a total of 170 clinical samples and 15 sewage samples for SARS-CoV-2 whole-genome sequencing, and the sequences have been submitted to GISAID (EPI_ISL_20102110–EPI_ISL_20102275). To obtain the sequence information of SARS-CoV-2, we used an amplicon-based enrichment approach to prepare the sequencing library. The reverse transcription and amplification steps were conducted using the ULSEN^®^ 2019-nCoV Whole Genome Kit (Micro-Future, Beijing, China). A total of 16 μL of viral RNA was used to reverse transcription, resulting in the synthesis of the first-strand cDNA. Subsequently, primer pools A and B were used to amplify the viral genome. The PCR product was purified using AMPure XP beads (Beckman Coulter, Brea, CA), and the concentration was adjusted to 0.2 ng/μL. Paired-end libraries were generated following the reference guide of the Nextera XT DNA Library Preparation Kit (Illumina, San Diego, CA). To enable multiplexing, the samples were indexed using the Nextera XT index kit (Illumina, San Diego, CA). For library quantification and validation, we used the Qubit 4.0 Fluorometer system (Life Technologies, Carlsbad, CA) and the 2100 Bioanalyzer (Agilent Technologies, Santa Clara, CA). The library was sequenced on the NextSeq 2000 platform using the NextSeq 1000/2000 P1 Reagents Kit (Illumina, San Diego, CA), with a run length of 300 cycles.

### 2.5 Results validation and quality control

To ensure the accuracy of results and prevent cross-contamination during sample collection, transportation, and testing, both whole program blank sample and laboratory blank sample were included in the analysis. The whole program blank sample was prepared by placing saline into a sample container, which was then subjected to the same procedures as real samples, including transportation to the sampling site, environmental exposure, collection, storage, transportation, and laboratory testing. The laboratory blank was also prepared using saline and processed alongside real samples during laboratory procedures. During RT-qPCR detection, a standard sample with a known concentration (ORF 1a/b: 7.13 × 10^5^ copies/mL, N: 7.8 × 10^5^ copies/mL), as well as positive and negative controls provided in the test kit, were included to verify the accuracy of the results. If the measured concentration of the standard sample deviated less than 5% from the known value, the results were used for further analysis. Similarly, for clinical samples, positive standards, saline, and positive/negative controls within the kit were included in each test.

To minimize cross contamination, genomic amplification and library preparation of clinical and sewage samples were performed separately using a Sciclone G3 NGSx iQ liquid handling workstation (Revvity, Waltham, MA). Sequencing of sewage and clinical samples was also carried out separately to avoid contamination

### 2.6 Data analysis

Post-sequencing analysis of the raw data, including quality assessment, trimming, removal of ambiguous bases, and amplification primer removal, was initially performed using the MicronCoV® Analyzer software (Micro-Future, Beijing, China) with default settings. The resulting high-quality clean reads were used for downstream analysis. Due to distinct analysis purposes, different analytical strategies were employed for clinical samples and sewage samples.

For clinical samples, we followed the analytical method proposed by Sun et al. (2022): Clean reads were aligned to the reference genome NC_045512.2 (downloaded from the National Center for Biotechnology Information) using BWA-MEM v0.7.17 with default parameters (Li, 2013), BAM file sorting was performed using Samtools v1.17 (Li et al., 2009), and PCR duplicate removal was carried out using the Genome Analysis Toolkit (GATK) v4.2.12.0 (McKenna et al., 2010). The consensus genome sequences were obtained using Ivar v1.4.2 (Grubaugh et al., 2019), with regions of sequencing depth less than 5 masked as “N.” sequences with genome coverage exceeding 96% were retained for downstream analysis. SARS-CoV-2 lineage classification was performed using Nextclade,^2^ based on the latest clade reference database at the time of analysis.

For sewage samples, we followed the pipeline available on GitHub^3^: Clean reads were aligned to the same reference genome NC_045512.2 using Minimap2 v2.26-r1175 with default settings (Li, 2018). After sorting the BAM file using Samtools (Li et al., 2009), we conducted a deconvolution analysis using the Freyja v1.3.12 to estimate the relative abundance of SARA-CoV-2 lineages present in each sewage sample (Karthikeyan et al., 2022).

## 3 Results

### 3.1 Monitoring results

**Figure 1:**
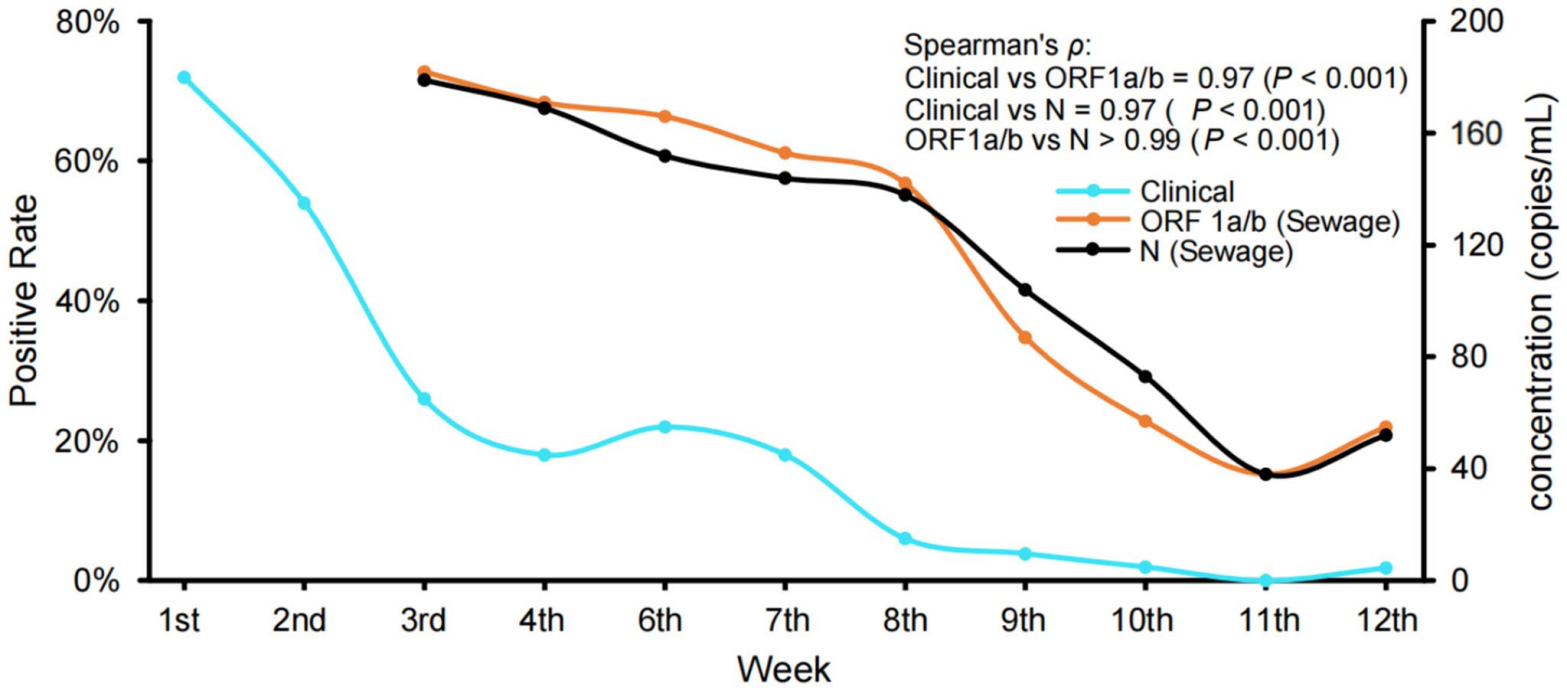
Variations in the rate of clinical positive and the concentration of SARS-CoV-2 RNA in sewage samples. The clinical positive rate was calculated based on the proportion of SARS-CoV-2 positive patients identified by RT-qPCR targeting ORF1a/b and N genes from respiratory specimens. Monitoring work was briefly halted during the 5th week, which coincided with the Chinese New Year. Spearman’s correlation between sewage RNA concentration (ORF1a/b and N genes) and clinical positivity rates was strong and statistically significant for both targets (*ρ* = 0.97, *p* < 0.001). The two gene targets also showed a strong correlation with each other (*ρ* > 0.99, *p* < 0.001).

RT-qPCR was performed on all 375 sewage samples and 614 clinical samples, and the positive rate of sewage samples was 81.6%. To compare sewage surveillance with clinical surveillance, we evaluated the temporal trends in SARS-CoV-2 RNA concentrations (copies/mL) in sewage alongside the weekly positive rate of clinical samples (Figure 1). Both ORF1a/b and N gene concentrations in sewage showed strong correlations with clinical positivity rates (Spearman’s *ρ* = 0.97, *p* < 0.001). These results showed that the changes in the concentration of SARS-CoV-2 RNA in sewage samples were consistent with the dynamic trend of clinically monitored cases. Moreover, the high correlation between ORF1a/b and N gene targets (*ρ* > 0.99, p < 0.001) supports the reliability and internal consistency of SARS-CoV-2 quantification in sewage samples. In addition, we found that after the 8th week, the number of copies of SARS-CoV-2 RNA in the sewage decreased rapidly to less than 100 copies/mL. It is very hard to obtain actual sequencing information from samples with such high Ct values using the current experimental protocol. Therefore, sequencing was not performed on samples collected after the 8th week. As a result, sequencing data were available only for the first 5 weeks, during which the viral loads were sufficient for reliable analysis. Despite covering only 5 weeks, this provided adequate data for further analysis.

### 3.2 Deconvolution of SARS-CoV-2 lineages in sewage samples versus clinical monitoring

**Figure 2:**
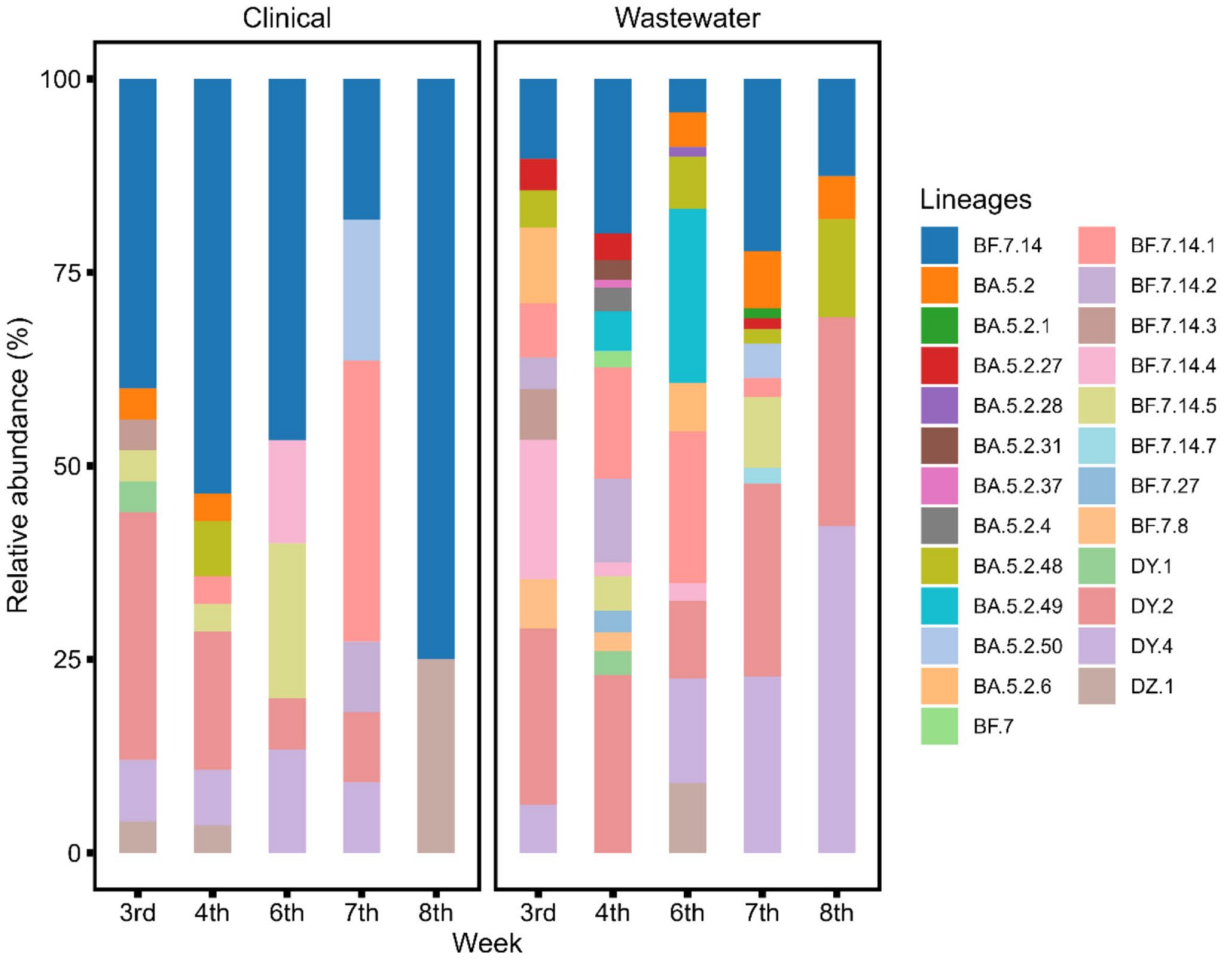
Sample deconvolution shows the relative abundance of SARS-CoV-2 lineages in clinical and sewage samples at the same time point. A significantly higher number of SARS-CoV-2 lineages were detected in sewage compared to clinical samples (paired *t*-test, *t* = 6, df = 4, *p* < 0.05).

The data analysis processes for sewage samples and clinical samples were different. Each sewage sample contained multiple SARS-CoV-2 lineages. Through deconvolution, we could get the proportion of each lineage. We averaged the abundance of each lineage across multiple sewage data over 1 week. The results were then compared with the analyses of clinical samples collected during the same period (the 3rd to the 8th week of 2023) (Figure 2). All sewage detections correspond to an estimated VOC prevalence of at least 1%. We observed that the sewage samples contained a greater number of lineages than those identified in the clinical monitoring. The abundance of SARS-CoV-2 lineages in sewage samples offered a broader perspective on the viral diversity within the community compared to the clinical samples. This suggests that sewage surveillance could be a valuable tool for monitoring the emergence and prevalence of various SARS-CoV-2 lineages, thereby contributing to a more comprehensive understanding of the viral landscape.

### 3.3 Longitudinal prevalence of select SARS-CoV-2 lineages in sewage and clinical data

**Figure 3:**
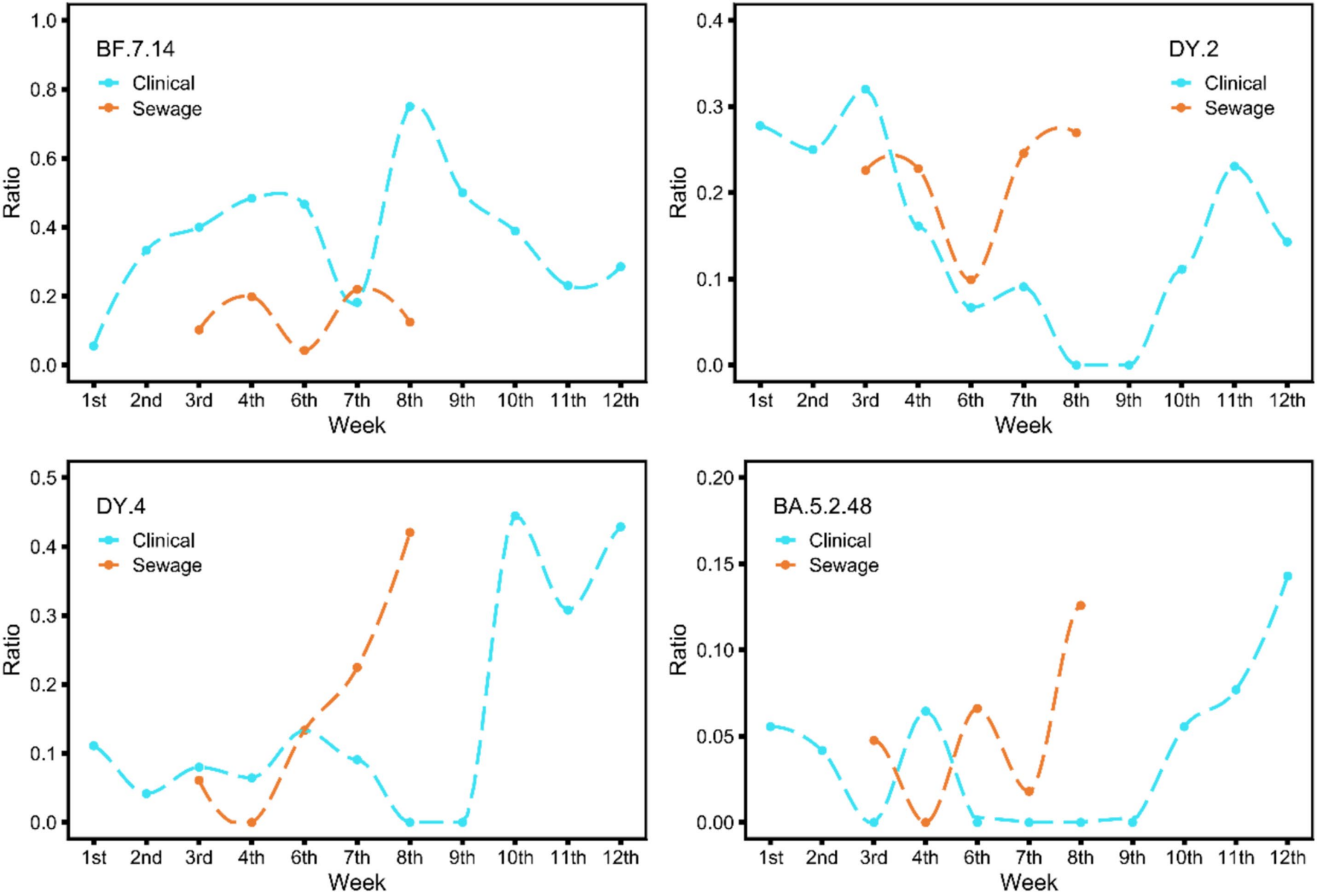
The figure shows the variations in the epidemic trend seen in clinical and sewage samples over the study period for four widely prevalent lineages (BA.5.2.48, BF.7.14, DY.2, DY.4). The cyan line represents the epidemic trend of clinical samples; the monitoring time is from the 1st week to the 12th week of 2023. The red line represents sewage samples; the time is from the 4th week to the 9th week of 2023. These plots were generated using the R package *ggplot2*, and smoothed curves were fitted using the *loess* method.

To compare the prevalence trends of specific lineages in sewage and clinical samples, we selected lineages that have existed in clinical samples for at least 6 weeks for analysis because it is impossible to accurately determine the change trend of lineages that have existed for too short a time. There were a total of 4 lineages, including BA.5.2.48, BF.7.14, DY.2 (BA.5.2.48.2), and DY.4 (BA.5.2.48.4) (Figure 3). The figure illustrates that the various lineages displayed different prevalence patterns. The trend of BF.7.14 in sewage samples was similar to that observed in clinical samples, but the change was 1 week earlier than in clinical samples, and another notable note was that the abundance in sewage was much lower. The numerical abundance values of the other three types in sewage samples were comparable to those in clinical samples, and the patterns of increase and decrease were similar to those in the clinical samples. In clinical samples, these three lineages showed a noticeable increase in the 9th week after a period of fluctuation. Moreover, the proportions of these three lineages also increased rapidly in the sewage samples, although the starting time of each lineage occurred at different points in time: DY.2 began in the 6th week, DY.4 in the 4th, and BA.5.2.48 in the 7th, all preceding the clinical start time.

## 4 Discussion

There is growing evidence for the presence of SARS-CoV-2 RNA in feces and municipal sewage, providing not only a warning signal for potential environmental contamination but also supporting the feasibility of early epidemic detection through sewage monitoring (Yousif et al., 2023; Kadam et al., 2025; Ahmed et al., 2020). SARS-CoV-2 is typically excreted in feces during the early stages of infection, often before the onset of symptoms (Wu et al., 2022; Moura et al., 2022). This feature enables the virus to be captured in sewage systems, making sewage a valuable tool for monitoring the community transmission of SARS-CoV-2. Recent studies have shown that sewage can be effectively used for real-time surveillance, especially where clinical testing is limited (Perez-Zabaleta et al., 2025; Islam et al., 2023).

The current SARS-CoV-2 epidemic prevention and control has entered a new stage. We conducted urban sewage SARS-CoV-2 surveillance in a standardized and organized manner using RT-PCR and high-throughput sequencing technologies in order to monitor and assess the epidemic intensity and characteristics of SARS-CoV-2 infections. From the 1st to the 12th week of 2023, we collected 375 sewage samples and 614 clinical patient samples from Yantai City, Shandong Province, China, for this study. The levels of SARS-CoV-2 in sewage samples were determined by quantitative PCR analysis and compared with clinical samples. The results showed that the copies of SARS-CoV-2 RNA in sewage were closely correlated with the rate of positive clinical tests, consistent with meta-analyses confirming that SARS-CoV-2 concentrations in sewage reflect community infection trends (Li et al., 2023). These findings demonstrate that sewage can serve as a reliable signal for outbreak surveillance, particularly in early outbreak detection.

Whole viral genomes are used in genomic surveillance, which, in contrast to RT-qPCR-based variation surveillance, can identify the SARS-CoV-2 strains that are circulating in the public and identify potential routes of transmission for infected individuals. High-throughput sequencing of sewage can be used to track the spread and dissemination of new and existing variants across communities (Kumar et al., 2020; Ahmed et al., 2020; Rothman et al., 2021). However, genomic detection in sewage poses technical challenges. RNA sequencing coverage is low in complex environmental samples due to factors such as low viral load, severe RNA fragmentation, and PCR inhibitors (Baaijens et al., 2021). To test the practicality of sewage genome monitoring in studying virus transmission in communities, we explored methods and conditions for high-throughput sequencing of SARS-CoV-2 in sewage. We obtained nearly complete virus genomes from sewage samples with a cycling threshold (Ct) below 32 (median: 91.27%). Admittedly, it is a challenging task to obtain virus information from samples with higher Ct values.

During this study period, we observed dynamic epidemics of BF.7, BF.5.2, and their subclades. By comparing the changes in the abundance of SARS-CoV-2 lineages in sewage and clinical samples, we found that more lineages could be detected in sewage samples than in clinical samples. This reflects the complex nature of sewage viral material. As reported in other regions, sewage surveillance captures a broader snapshot of viral diversity, including low-prevalence or emerging variants that might not be detected through routine clinical testing (Smyth et al., 2022). As an information-dense resource for estimating the prevalence of specific viral lineages, providing information not only on overall infection dynamics but also on the rise and fall of specific VOCs. Among the more widespread lineages, the abundance changes in sewage samples were consistent or highly similar to those in clinical samples. For the BF.7.14 subtype, the magnitude of abundance in sewage was considerably lower, indicating that each lineage may possess its own distinct features. It cannot be ruled out that the limitations of the detection range lead to elevated clinical outcomes. This issue still needs to be studied and confirmed in follow-up monitoring. Combined with the abundance changes of four lineages, sewage is more sensitive to the growth of dominant strains, and under existing monitoring strategies, the rapid growth of dominant strains can be predicted at least 1 week in advance.

In conclusion, our study highlights the potential of sewage surveillance as a robust tool for monitoring SARS-CoV-2. The detection of SARS-CoV-2 in sewage not only serves as an early warning system for potential outbreaks but also provides a cost-effective and extensive method for tracking the prevalence and diversity of viral lineages within a community. This approach offers a novel perspective for epidemiological research and has significant implications for public health decision-making. We believe that virus testing in sewage will play an important role in public health and help us better respond to epidemics of known and even unknown viruses.

## Data Availability

The original contributions presented in the study are included in the article/supplementary material, further inquiries can be directed to the corresponding authors.

## Footnotes

^1^ https://data.who.int/dashboards/covid19/cases

^2^ https://clades.nextstrain.org/

^3^ https://github.com/niemasd/SD-COVID-Sequencing
